# In vivo experimental validation of detection of gastric slow waves using a flexible multichannel electrogastrography sensor linear array

**DOI:** 10.1186/s12938-022-01010-w

**Published:** 2022-06-27

**Authors:** Atchariya Sukasem, Stefan Calder, Timothy R. Angeli-Gordon, Christopher N. Andrews, Gregory O’Grady, Armen Gharibans, Peng Du

**Affiliations:** 1 University of Auckland, Auckland, New Zealand; 2 The Cumming School of Medicine, University of Calgary, Calgary, Canada

**Keywords:** EGG, Electrophysiology, High-resolution mapping, Gastric slow waves

## Abstract

Cutaneous electrogastrography (EGG) is a non-invasive technique that detects gastric bioelectrical slow waves, which in part govern the motility of the stomach. Changes in gastric slow waves have been associated with a number of functional gastric disorders, but to date accurate detection from the body-surface has been limited due to the low signal-to-noise ratio. The main aim of this study was to develop a flexible active-electrode EGG array. Methods: Two Texas Instruments CMOS operational amplifiers: OPA2325 and TLC272BID, were benchtop tested and embedded in a flexible linear array of EGG electrodes, which contained four recording electrodes at 20-mm intervals. The cutaneous EGG arrays were validated in ten weaner pigs using simultaneous body-surface and serosal recordings, using the Cyton biosensing board and ActiveTwo acquisition systems. The serosal recordings were taken using a passive electrode array via surgical access to the stomach. Signals were filtered and compared in terms of frequency, amplitude, and phase-shift based on the classification of propagation direction from the serosal recordings. Results: The data were compared over 709 cycles of slow waves, with both active cutaneous EGG arrays demonstrating comparable performance. There was an agreement between frequencies of the cutaneous EGG and serosal recordings (3.01 ± 0.03 vs 3.03 ± 0.05 cycles per minute; *p* = 0.75). The cutaneous EGG also demonstrated a reduction in amplitude during abnormal propagation of gastric slow waves (310 ± 50 µV vs 277 ± 9 µV; *p* < 0.01), while no change in phase-shift was observed (1.28 ± 0.09 s vs 1.40 ± 0.10 s; *p* = 0.36). Conclusion: A sparse linear cutaneous EGG array was capable of reliably detecting abnormalities of gastric slow waves. For more accurate characterization of gastric slow waves, a two-dimensional body-surface array will be required.

## Introduction

Motility (contractions) of the gastrointestinal (GI) tract involves interplay between myoelectrical activity (slow waves) and neurohumoral modulation [1]. Abnormal GI motility can lead to pervasive but poorly understood digestive disorders, such as gastroparesis and slow transit constipation [2–4]. Recent invasive high-resolution (HR) mapping techniques, employing a spatially dense array of up to 256 electrodes placed directly on the serosal surface of the stomach, enabled the analysis of slow wave activation sequences in accurate spatiotemporal details [5]. In the healthy state, human gastric slow waves originate from the pacemaker region in the proximal stomach and propagate as an antegrade wavefront towards the pylorus, culminating in multiple simultaneous wavefronts moving along the organo-axis of the stomach [6]. Persistent abnormal gastric slow waves were identified in patients suffering from motility disorders such as gastroparesis and chronic unexplained nausea and vomiting [7, 8].

While HR mapping provides the direct evidence of the role of gastric slow wave dysrhythmias in diseases, the invasive nature of the recording technique limits its application in a clinical setting [9]. While other minimally invasive techniques such as laparoscopic and endoscopic deployments have been proposed, both methods still require extensive preparation of the stomach and the subject being placed under general anesthesia or sedation [10, 11]. To overcome the inherent challenges associated with HR mapping and other minimally invasive recording techniques, cutaneous electrogastrography (EGG) has been utilized as a non-invasive tool that is suitable for routine deployment during clinical studies. Similar to the application of electrocardiography, EGG measures the resultant activity of gastric slow waves using cutaneous electrodes placed on the epigastrium of the torso. While EGG holds promising potential as a diagnostic tool for detecting gastric slow wave dysrhythmias associated with the common symptoms of digestive disorders [7, 12], there are a number of critical limiting factors that have prevented EGG from becoming widely adopted, these include: (i) few studies have investigated the detailed correlation between gastric slow waves and EGG [12–14]; (ii) most studies applied EGG to analyze the temporal information only [16], whereas changes in spatial characteristics were not investigated; and (iii) extensive skin preparation is required to reduce the skin–electrode impedance [9].

Active electrodes are a common approach to maximize the input impedance while minimizing the output impedance, presenting a solution to power interference and lower requirement for electrode–skin contact [17, 18]. There are a number of active electrode designs and configurations, with some involving additional components on the electrode to provide filtering and signal gain, while others can be unity gain, i.e., buffer electrodes. A number of two-wired designs have been proposed to further reduce the size of the active electrodes, while flexible circuitry design has also been adopted to further compact the size of the wires [17, 19]. The potential advantages of active electrodes are of particular relevance to EGG applications where the recordings are generally taken over a long period (up to hours) at a time [6]. To our best knowledge, a comparative active EGG electrode design and validation has not been investigated.

The main objective of this study was to develop a flexible cutaneous EGG array for the detection of gastric slow waves, by combining active electrodes and flexible circuits for small scale design. The EGG electrodes were evaluated in a benchtop environment before validated against HR mapping directly from the stomach in a series of acute pig studies, to determine the effects of spatial variation of gastric slow waves on cutaneous recordings using the novel platform. The outcome will inform future application and analysis of EGG signals.

## Results

### Benchtop test results

**Fig. 1:**
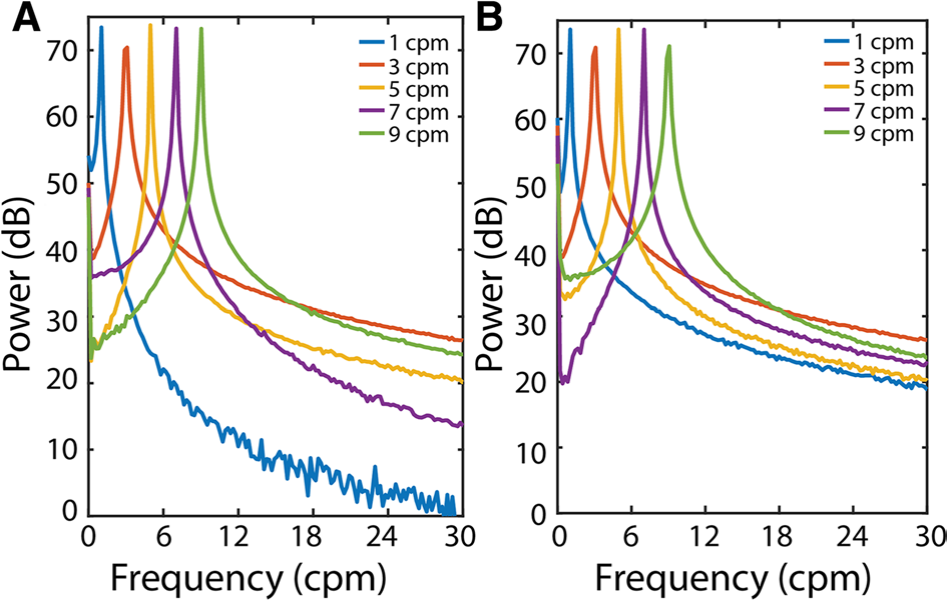
Benchtop test results of two opamps for EGG recordings in response to synthetic sinusoidal signals at 1, 3, 5, 7 and 9 cpm. **A** TLC272BID and (**B**) OPA2325 had a similar high power of 70 dB at 3 cpm, low mains interference of − 4 dB, but the harmonic interference at 100 Hz in TLC272BID (− 14 dB) is higher than it in OPA2325 (− 20 dB)

The two chosen opamps demonstrated adequate signal-to-noise ratio values over the common range of frequencies present in an EGG signal as shown in Fig. 1. In particular, the TLC272BID opamp demonstrated an average SNR of 73.8 dB ± 0.5 dB over 1–9 cpm, an average mains power of − 2.5 dB ± 1.2 dB at 50 Hz, and an average harmonic power of − 13.2 dB ± 0.9 dB at 100 Hz. On the other hand, the OP2325 opamp demonstrated an average SNR of 73.8 dB ± 0.5 dB over 1–9 cpm, an average mains power of − 0.8 dB ± 1.8 dB at 50 Hz and an average harmonic power of − 15.1 dB ± 2.185 dB at 100 Hz. The difference in the 1 cpm signal response was potentially due to a combination of the lower CMRR, higher offset and greater gradient of the equivalent input noise of the TLC272BID, which caused its gain at the 1 cpm signal to be less consistent than the OPA2325 (Fig. 1).

The two opamps demonstrated similar levels of performance during the bench top tests, but the OPA2325 opamp had a higher average floor power than the TLC272BID opamp in 0–125 Hz (− 20.63 dB ± 0.02 dB vs − 22.77 dB ± 0.02 dB; *p* < 0.001). Overall, the gains of the TLC272BID and OPA2325 opamps were similar over the 1–9 cpm range (1.004 ± 0.004 vs 1.000 ± 0.004; *p* > 0.36). Noticeably, the TLC272BID opamp demonstrated a slightly higher gain than the OPA2325 opamp at 1 cpm (1.05 vs 1.00), though the relative size of the gain is minor.

### In vivo validating results

**Fig. 2:**
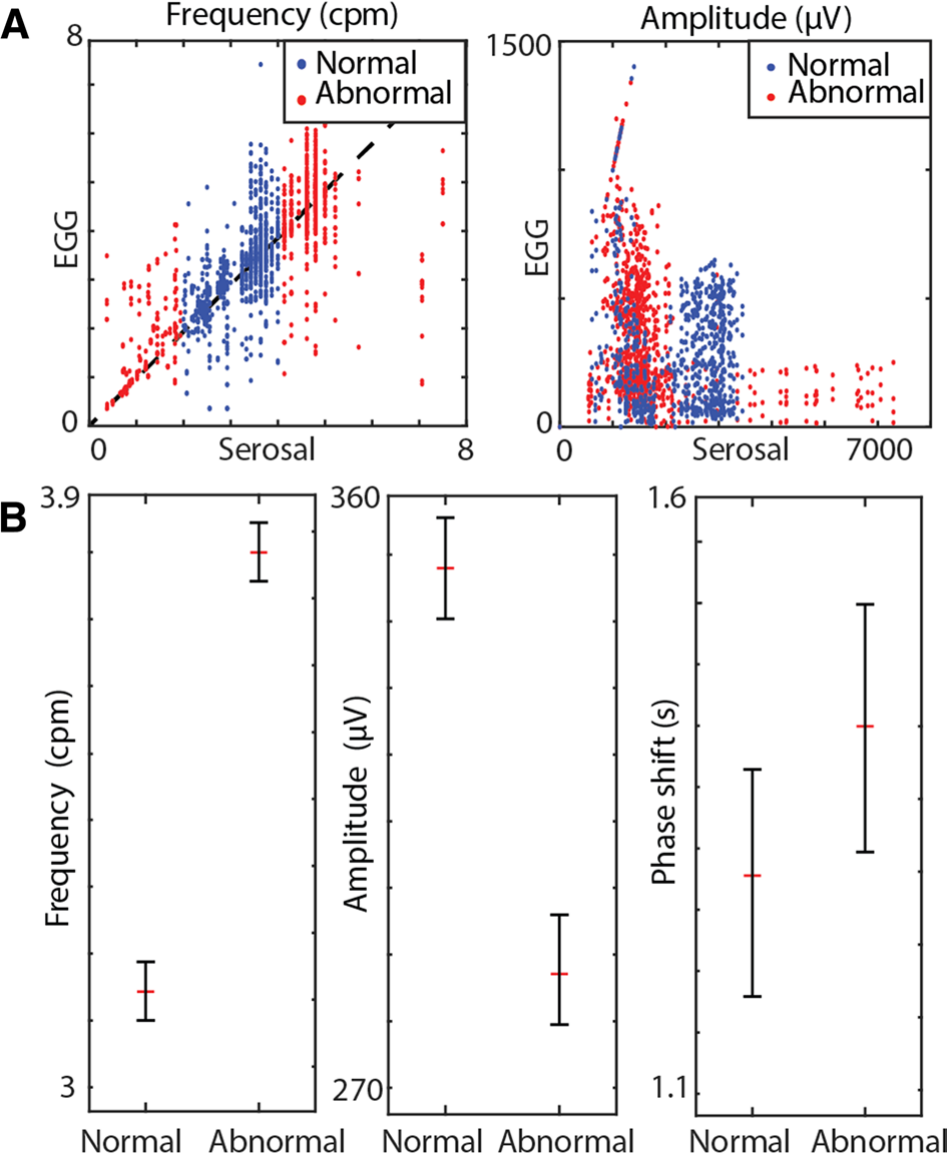
Summary of cutaneous EGG correlation with serosal recordings. **A** Correlations between frequencies (*R*^2^ = 0.641) and amplitudes (*R*^2^ = 0.011). **B** Comparison of cutaneous EGG characteristics (frequency, amplitude, and phase-shift) between normal and abnormal slow waves based on serosal recording classifications

Serosal HR mapping across the ten pigs exhibited both regular and irregular gastric slow waves from the anterior of the stomach, with both antegrade and dysrhythmic propagations matching the classifications of the previously established criteria [20]. Overall, all pigs demonstrated normal gastric slow waves, while seven pigs also exhibited episodes of spontaneous abnormal propagations. There was a change in the amplitude of the serosal recordings between normal and abnormal slow waves (1.71 ± 0.03 mV and 2.25 ± 0.03 mV; *p* < 0.001) and a similar trend was observed on in the cutaneous EGG recordings (Fig. 2B).

A total of 26 sessions of recordings were performed, out of which 11 were recorded using the flexible arrays embedded with the TLC272BID array, and 15 were recorded using the flexible arrays embedded with the OPA2325 array. After time-synching and allowing for the removal of 5 min of recording from each session to account for the edge effects of the CWT analysis, a total of 709 cycles of slow waves were analyzed (on average 35.5 ± 28.3 cycles per subject), out of which 314 cycles were recorded using the TLC272BID array and 395 cycles were recorded using the OPA2325 array.

As frequency is the standard metric of conventional EGG measurement [22, 23], the temporal match between individual channels in the EGG electrode array and serosal data was analyzed first, as shown in Fig. 2A. There was an agreement between the frequencies of the serosal and the combined cutaneous EGG recordings of both opamps (3.01 ± 0.03 vs 3.03 ± 0.05; *p* > 0.75), as well as a high degree of correlation between the frequency reported by the cutaneous EGG and serosal recordings (*R*^2^ = 0.64), where no such correlation existed between the amplitudes reported (*R*^2^ = 0.01), as shown in Fig. 2B.

When compared to serosal recordings separately, the two cutaneous EGG arrays also demonstrated comparable performance. The TLC272BID array detected a frequency of 3.42 ± 0.07 cpm compared to the serosal frequency of 3.30 ± 0.05 cpm (*p* > 0.14), whereas the OPA2325 EGG array detected a frequency of 3.27 ± 0.04 cpm compared the to the serosal frequency of 3.22 ± 0.07 cpm (*p* > 0.54).

Based on the characterization of slow waves from serosal recordings, the frequency of cutaneous EGG during normal antegrade propagation was lower than the frequency of EGG during abnormal propagation (3.14 ± 0.04 vs 3.83 ± 0.05 cpm; *p* < 0.01), as shown in Fig. 2B. The amplitude of cutaneous EGG also demonstrated significant difference between normal propagation and abnormal propagation (348 ± 7 vs 288 ± 8 µV; *p* < 0.01). Finally, there was no statistical difference between the average phase-shift between the cutaneous electrodes during normal and abnormal propagations (1.28 ± 0.09 vs 1.40 ± 0.10 s; *p* = 0.36).

**Fig. 3:**
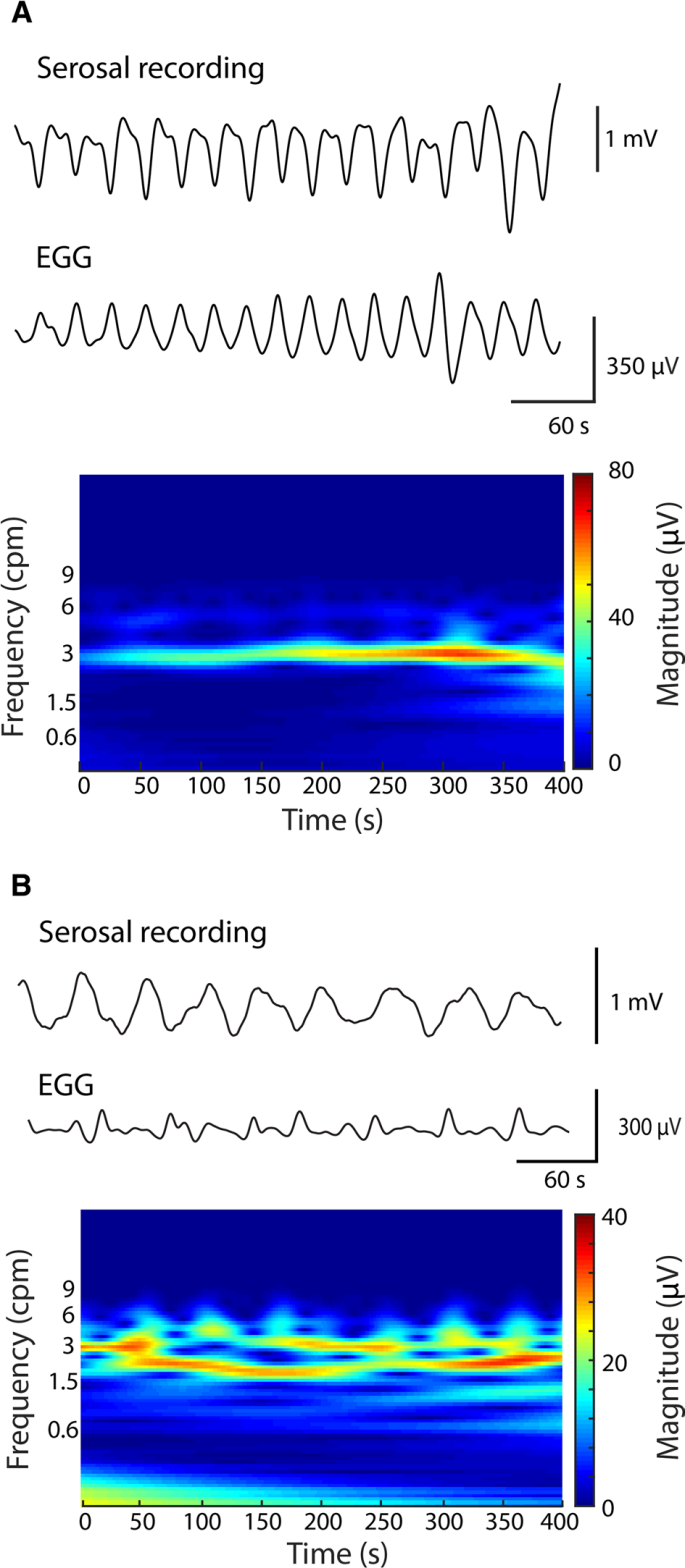
**A** Normal slow waves: the cutaneous EGG shows consistent 3 cpm slow waves corresponding to its slow waves in the serosal recording, where the CWT plot showed steady 3 cpm slow waves. **B** Abnormal slow waves: the cutaneous EGG represents abnormal slow waves (unsteady frequency) in the serosal recording, where the CWT plot showed abrupt changes in slow wave frequency over the period of the recordings

Two examples of simultaneous serosal and cutaneous EGG recordings are demonstrated in Fig. 3, which were captured in all ten subjects studied. In the first instance when the slow wave activity was stable over a period of 400 s (Fig. 3A), the corresponding cutaneous EGG from one of the channels on the active array showed a one-to-one match to the serosal recording. The corresponding CWT of the cutaneous data illustrated a clear and stable high signal band at 3 cpm (310 ± 50 µV). In the second instance when the frequency of the serosal recording shifted to the bradygastria range of 1.38 ± 0.27 cpm (Fig. 3B), there was also a corresponding reduction in the frequency of cutaneous EGG, while maintaining the one-to-one match to the serosal recording. Due to the variation in frequency during bradygastria, the CWT of the cutaneous signals also demonstrated a “scattering” of the dominant frequency band, which mostly stayed at approximately 1.5 cpm (277 ± 9 µV), but occasionally also switched back to 3 cpm, e.g., at 167 s.

**Fig. 4:**
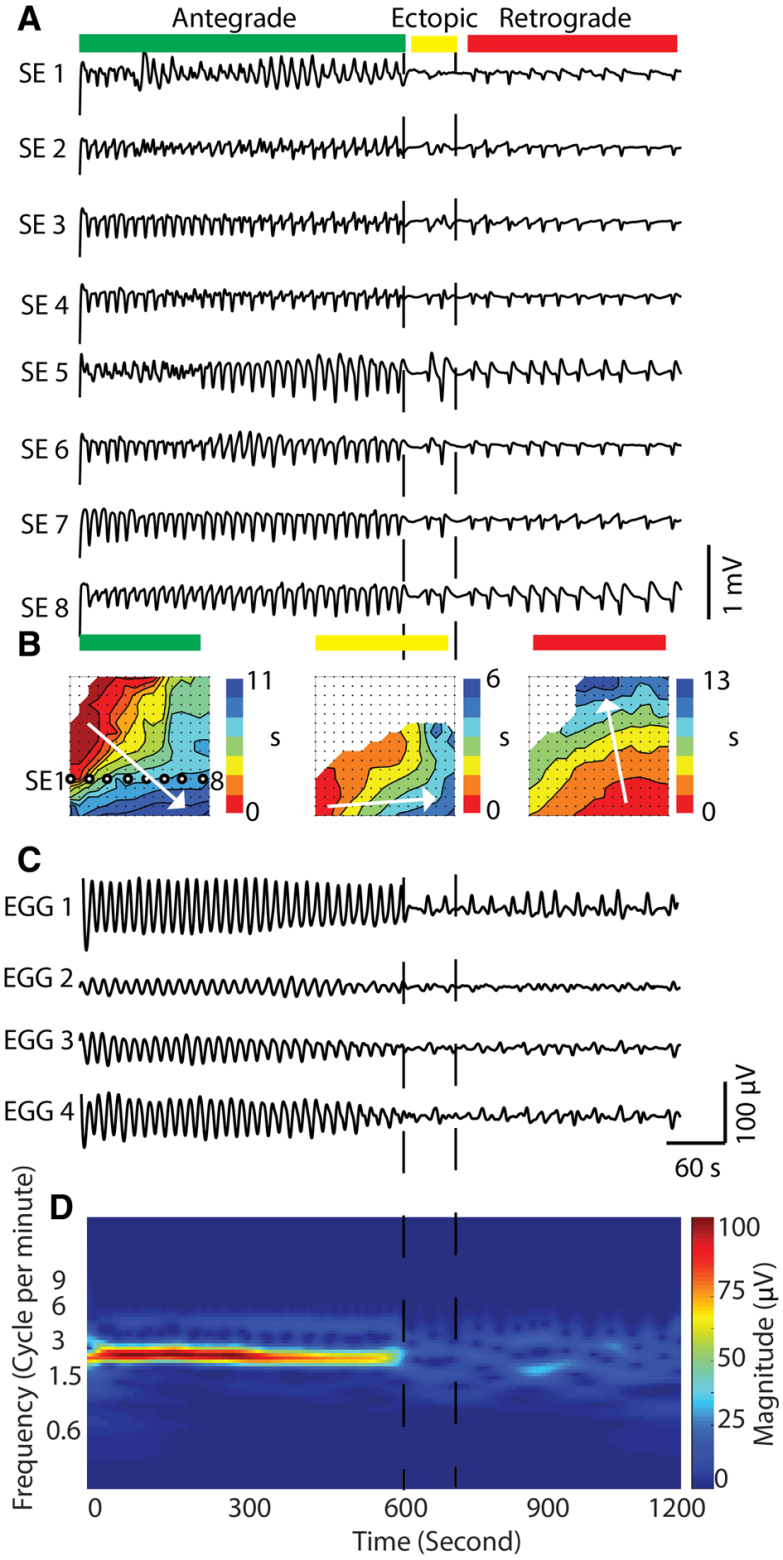
An example of corresponding slow waves in the serosal electrode and cutaneous EGG: **A** the traces from eight serosal electrodes in the same column are shown. The recording showed both normal and abnormal slow waves where the normal slow waves showed a frequency of 3 cpm in the antegrade manner (0–600 s) and the abnormal slow waves showed spatiotemporal deviations from the nominal slow waves (600–1200 s); **B** activation maps showed an individual slow wave’s propagation: antegrade (left), retrograde and ectopic (middle) and retrograde manners (right). The green, yellow, and red bars indicate the types of the activation plot during the recording. **C** Cutaneous EGG recordings correspond to the serosal traces. **D** CWT plot represents the contribution of the slow waves in the frequency spectrum over the period of the recording

An instance of cutaneous EGG during a transition from normal to dysrhythmic retrograde propagation over a 20-min period is demonstrated in Fig. 4, which occurred in 30% (3/10) of the subjects studied. The instance was representative of the typical spontaneous transition from normal to abnormal activities in the dataset. The serosal HR mapping array was placed in the proximal corpus, and for the initial 650 s of the recording, gastric slow waves propagated in the antegrade direction towards the pylorus over 32 cycles. The activation map of the antegrade activity was labeled by the green bar in Fig. 4B. During the same period of antegrade propagation, cutaneous EGG demonstrated a corresponding stable activity occurring at a frequency of 2.97 ± 0.19 cpm with an amplitude of 122 ± 70 µV, as shown in Fig. 4C, D. Spontaneous spatial dysrhythmias occurred at 709 s, as shown in Fig. 4A, indicated by the yellow bar, which presented a transitional phase that lasted approximately 154 s. During this period, the frequency and amplitude of gastric slow waves reduced to 1.15 ± 0.35 cpm, which was also detected by the EGG (Fig. 4C). Direction of propagation was reversed compared to the normal period, switching to retrograde propagation, which was slightly faster than the antegrade propagation (5.27 ± 0.04 vs 6.65 ± 0.06 mm s^−1^; *p* < 0.01). In contrast, there was no phase difference between normal and abnormal EGG recordings (0.72 ± 0.06 vs 0.95 ± 0.12 s; *p* = 0.09).

Sustained retrograde propagation over 490 s followed the transition phase. There was a notable reduction in frequency to 1.52 ± 0.41 cpm, as well as amplitude in the both serosal and EGG recordings (Fig. 4C). The amplitude of the slow waves in the serosal recording was reduced from 2.64 ± 0.07 mV to 1.84 ± 0.11 mV (*p* < 0.01), while the amplitude of EGG also from 107 ± 4 µV to 50 ± 8 µV (*p* < 0.01). The velocity during this period (6.62 ± 0.07 mm s^−1^) was comparable to the transitional phase (*p* = 0.3) and still faster than the antegrade propagation (*p* < 0.01). There was also no phase-shift difference between normal and abnormal EGG recordings (0.72 ± 0.06 vs 0.75 ± 0.07 s; *p* ≈ 0.19).

## Discussion

This study demonstrated the design, manufacturing and validation of a flexible EGG array with active electrodes. The flexible EGG arrays were validated against benchmark recordings taken directly from the serosa of the stomach using high-resolution (HR) mapping arrays. Both chosen operational amplifiers (opamps), OPA2325 and TCL272BID, demonstrated comparable performances. The main finding was that the active EGG arrays were capable of reliably detecting the changes in the frequency of the underlying gastric slow waves, both during normal and abnormal activations. In addition, changes in frequency, rather than amplitude of gastric slow waves, had the most significant impact on the amplitude of EGG signals.

While a number of previous studies have proposed the use of active electrodes for cutaneous EGG recordings [23, 24], very few have incorporated the circuitry into a flexible design with validation against serosal recordings during both temporal and spatial changes of gastric slow waves. To complicate the matter further, in conventional EGG recordings, the term “active electrodes” has also been used to distinguish the difference between recording electrodes and the reference electrode [25]. The recording electrodes in most cases were usually Ag/AgCl passive electrodes, rather than an electrode with an in-built circuitry. While other investigators have potentially used commercial active electrode systems for recording EGG [26], the opamps were often not specifically tested to perform adequately near DC, which is required for EGG. Two additional opamps (OPA2211 and OPA388) were also considered for the application of active EGG electrodes, but were not incorporated into the design due to initially failing to obtain adequate signals at the benchtop testing stage, due to strong interferences by the power-mains and its harmonic. The common solution to the problem would be to eliminate the harmonic interference at the power pin of the opamps, by connecting it to a 0.1-µF ceramic bypass capacitor in parallel to the ground. While this would slightly increase the complexity of the circuitry, it would be worthwhile to comprehensively test the performance of more opamps for EGG applications.

The buffer electrode designs adopted in the present study offered the simplest onboard circuitry, but at the cost of potential power efficiency, and increased complexity at the backend of the acquisition system to deal with the DC offset [27]. In addition, while the flexible design offers a compact way to manage the size of the cables, more advanced two-wired configurations that have been proposed could have been incorporated to further reduce the size of the cables and simplify the onboard circuitry [17]. In future, more compact designs and compliant substrate materials should offer better adhesion and placement on the abdomen, especially for wearable applications [28]. More advanced materials such as PEDOT:PSS could also be deposited to create organic sensor arrays for more biocompatible recordings [29].

The choice of filter has been recognized as a critical setup in EGG signal processing [21]. Given gastric slow waves mainly occur around 3 cpm (0.05 Hz) range, a filter with a gradual roll-off and/or ripples in the passband would distort the EGG by allowing artifacts such as respiration, ECG and/or movements. The cutoff threshold implemented in the present study was based on double the nominal gastric slow wave frequency observed from the porcine subjects [30]. Another consideration was by acquiring EGG at sampling frequency of 250 Hz, if a cutoff threshold of 0.15 Hz (9 cpm) was applied, it would represent 0.12% of the Nyquist range, which could lead to stability problems with the filter. The higher bandwidth of the signal compared to the filter window may also impact the decision of the type of window-function that can be applied to signal prior to spectral analysis. Therefore, the present study adopted an iterative down-sampling approach by reducing the sampling frequency to 2 Hz first and then applying a low-pass Butterworth filter which has a flat passband with a cutoff that account for 15% of the Nyquist range.

Due to the non-invasive advantage of EGG a number of studies have attempted to address the correlation between gastric slow waves and cutaneous signals. Currently, the best evidence comes from simultaneous recordings from the serosal or mucosal surfaces of the stomach and cutaneous electrodes [32–38]. In general, the dominant frequencies of EGG obtained using FFT or CWT would be matched to the frequency of the underlying serosal recordings. The present study followed the same methodology and demonstrated that the active EGG arrays were capable of detecting the underlying gastric slow waves with identifiable features in the time domain (Fig. 3) in addition to relating the changes that were correlated to spatial variations (Fig. 4).

Notably the present study demonstrated that while there was a difference in the amplitudes of slow waves between normal and abnormal periods in the serosal recordings, there was no direct correlation between amplitudes of serosal and cutaneous EGG recordings (Fig. 2). The difference between normal and abnormal periods of slow waves could be explained by an emergence of circumferential propagation from an ectopic source or following conduction block [36]. On the other hand, the changes in cutaneous EGG amplitudes could also be influenced by the skin impedance and orientation/distance of the stomach relative to the EGG electrodes. A similar observation was previous reported by Mintchev et al. who demonstrated that gradual distention of the canine stomach via atropine and glucagon increased cutaneous EGG amplitudes [13]. In the pigs, the greater curvature of the stomach is generally closest to the abdomen surface, but in a few instances the posterior surface of the stomach was observed to be closest to the abdominal wall. While the placement of the EGG array relative to the stomach could be determined as the position and the organo-axis of the stomach were known, this is not the case without the guide of medical imaging when EGG is applied in clinical practice, which would typically be performed in the absence of intra-operative access. A number of investigators have proposed to use a standard placement based on the ratio of the distance between the anatomical landmarks on the abdomen, and/or a greater number of electrodes to map an area of the torso [11, 28].

The most important factor influencing cutaneous EGG amplitude was likely the significant reduction in the power associated with dominant frequency of EGG compared to the normal activity (Fig. 4). Furthermore, the abnormal period was associated with a “scattering” of frequencies that reflected a fluctuation in the temporal stability of gastric slow waves. An underlying mechanism that could explain a reduction in the amplitude of cutaneous EGG without significant reduction of amplitude of slow waves could be the change in the portion of active gastric tissue due to the change in frequency. As the frequency was reduced, there was likely a corresponding increase in the distance between the successive wavefronts, which could lead to a reduction in the portion of the stomach that was active and/or a fewer number of simultaneous wavefronts. Previous modeling studies have shown that changes in wavefront separation and number of wavefronts could have a significance impact on the dipole that is used to represent the net activation of the stomach, resulting in a corresponding change in the simulated cutaneous potentials [37]. It has also been proposed that a refinement, i.e., closer inter-electrode spacing, would possibly be able to distinguish between the separation of wavefronts from the body-surface [38].

There are a number of limitations in the present study. The amplitude of signal used in the benchtop tests was a magnitude higher than the expected amplitude of EGG, which could have biased the benchtop results. Motility measurements, i.e., muscular contraction, and meal response under general anesthesia could not be performed and related to cutaneous recordings, so the advantages of active electrodes could not be fully assessed. In future, non-invasive studies, motility of the stomach and EGG could be captured simultaneously using scintigraphy or MRI using an appropriate system. Subjects should also be subjected to a standard meal to test the response of the stomach to nutrient and volume load over a longer time period (> 3 h), which is required to assess the condition of the stomach [39]. The active electrode in combination with a RDL reference system may present an advantage over the passive electrodes due to the less stringent requirement of skin preparation, which may improve post-recording side-effects. In the present study, because the animals were mechanically ventilated, the exact rate of respiration was known and there was no additional bodily movement artifact. In practice, on awake patients both the rate of respiration and bodily movements would introduce additional variable noise to the recordings. Effects of postures on EGG, such as standing up and crouching/flexing the torso could be tested. Other than encouraging the subject to stay still, additional measurements using an accelerometer on the acquisition system could be used either as a signal quality check or as a source to separate noise from signal using the Kalman filter. Filters and sampling frequency should also be considered for potential capture of “spikes” activity in the GI tract [40]. Theoretical mathematical characterization and analysis could also be performed to better justify the configuration of the opamps and design of appropriate filters to best reject artifacts while retaining the key information from EGG. Finally, the linear EGG array could be expanded to a 2D array to cover an area of the abdomen surface so more accurate phase analysis could be conducted.

## Conclusions

The main outcome of this study was the validation of an active-electrode linear array for cutaneous EGG, correlated with simultaneous serosal high-resolution mapping results. The array was shown to adequately detect the change in frequency of gastric slow waves and potentially present a wearable solution for chronic recordings. However, it is also clear that the sparse electrode configuration was more sensitive to changes in frequency than spatial changes in slow wave propagation, which should be recorded using 2D multichannel arrays that cover a significant portion of the epigastric region on the abdomen [41].

## Methods

### Operational amplifier selection and benchtop testing

Two CMOS operational amplifiers (opamps): OPA2325 and TLC272BID (Texas Instruments, TX, USA) were identified as appropriate for buffer EGG electrodes due to their characteristics listed in Table 1. These characteristics were deemed important in order to maximize the signals of interest at the targeted gastric slow wave frequencies while minimizing the supply current at safe levels from electric shocks and additive noises.

**Table 1:** Datasheet characteristics of OPA2325 and TLC272BID operational amplifiers

Electrical properties	OPA2325	TLC272BID
Input impedance	10^12^ Ω	10^12^ Ω
Output impedance	180 Ω	750 Ω
Offset voltage	± 150 μV	± 500 μV
Power consumption	4mW	16 mW
Common mode rejection ratio	114 dB	84 dB
Input voltage noise density	9 nV/√Hz	25 nV/√Hz
Unity gain stability	Yes	Yes

The frequency response of each opamp was quantified in a benchtop environment to demonstrate whether it was suitable for capturing the major frequency components [1–9 cycles per minute (cpm)] of typical EGG signals [20]. An input terminal of each opamp was directly connected to an output channel of a signal generator (AFG3000C Arbitrary/Function Generator, Tektronix Company, USA). The opamps were powered by ± 2.5 V by a DC power supply (Keithley-2231A-30-3, Tektronix Company, USA). The input signal was a 10 mV peak-to-peak sinusoidal wave with a frequency of 1 to 9 cpm with 0 mV offset. At each frequency, a 5 min stabilization period was taken for each recording. An output channel of the opamps was connected to an input of a Cyton biosensing board (OpenBCI, USA) via a breadboard and female–male jumper wires. Data were transferred to a laptop via a wireless USB dongle using customized acquisition software, OpenBCI GUI at a sampling frequency of 250 Hz. The signal-to-noise ratio (SNR) of the benchtop signals were quantified in power spectral density of the signal of interest, i.e., 1–9 cpm (*P*_signal_), over the 0–125 Hz band (*P*_noise_) using the formula below [42],

$${\text{SNR}}=20{\mathrm{log}}_{10}\frac{{P}_{\text{signal}}}{{p}_{\text{noise}}}.$$

### Flexible EGG electrode design

**Fig. 5:**
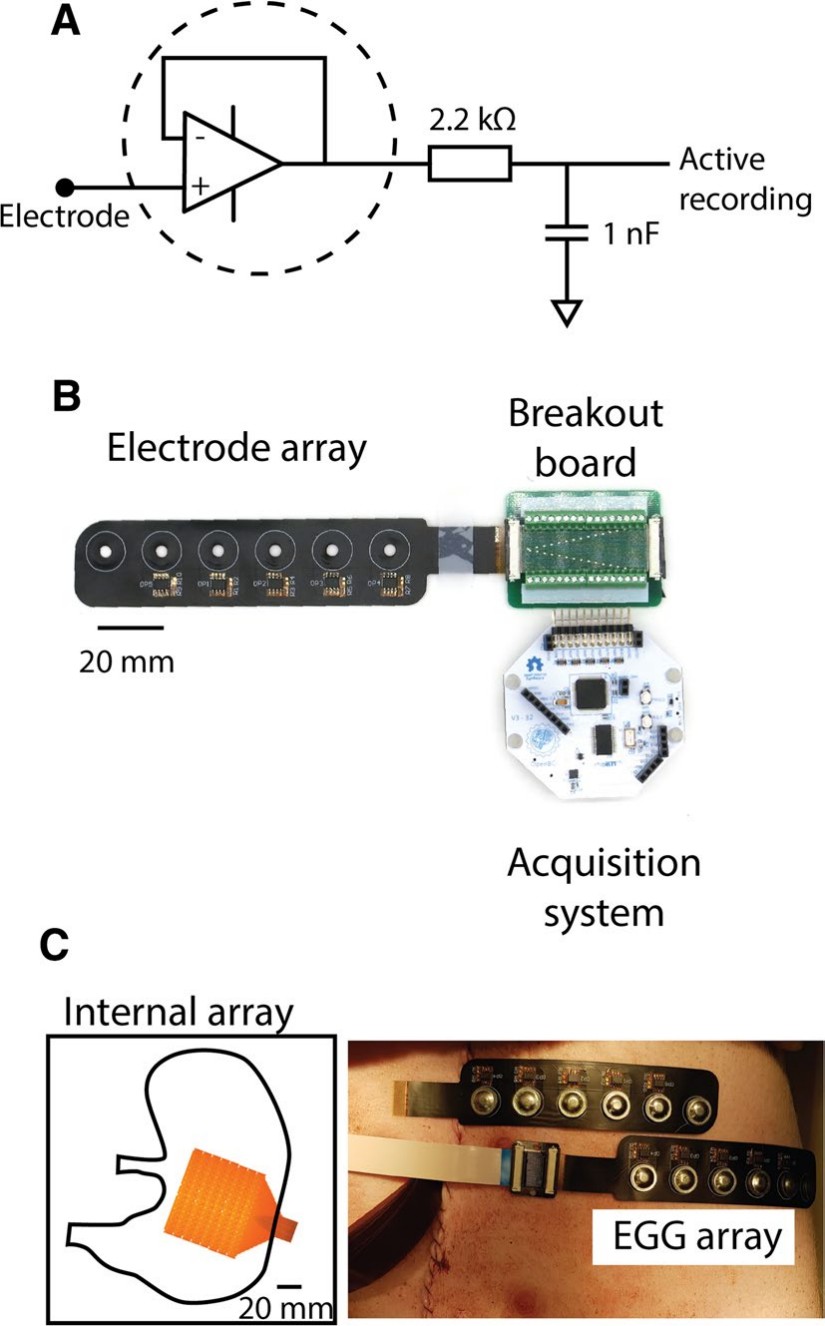
EGG amplifier, flexible array and animal validation setup. **A** The schematic drawing of the circuitry connection between the electrode mapping array and the Cyton biosensing board. **B** The main components of data acquisition, comprised an electrode array with four sensing buffer electrodes, and a common-mode sensing (CMS) and driven right-leg (DRL) referencing system. **C** The placement of the cutaneous active electrode array on the epigastrium and the internal HR mapping electrodes surgically placed on the serosal surface of the stomach. The orientation and coverage of the HR mapping electrode (16 × 16 electrodes; 4 mm inter-electrode spacing) relative to the stomach is shown on the left. The stomach is not drawn to scale

The buffer electrodes were incorporated in a flexible linear array design, with six electrodes at 20 mm inter-electrode spacing, as shown in Fig. 5. The DRL and four recording electrodes were active electrodes, occupying the four electrodes on the right-hand side of the array toward the connector end, while ground, located near the left-hand side, was passive. This configuration also allowed the DRL and reference electrodes to be placed further away from the stomach relative to the recording electrodes. The electrode array was made of polyimide flex and wrapped up with HCF-6000G for EMI shielding film. It was designed in order to integrate with a disposable ECG electrodes (Kendall™/Covidien Medi-Trace^®^ 200 Series, Medi-trace Inc., USA) through a 4 mm diameter hole using a tinned snap-connector, as shown in Fig. 5B (top). FR4 Stiffeners were added to the bottom layer to protect the integrity of the circuitry in the top layer. The hydrogel on the ECG electrodes generally provided enough adhesion to the skin and extra medical tapes were used to further secure the electrodes in place.

The buffer electrodes were supplied by ± 2.5 V by the Cyton biosensing board. The supply, return and signal leads of each electrode were routed via a custom PCB adapter with a 32-way ZIF connector to the Cyton biosensing board, as shown in Fig. 1C. Data were transferred to a laptop via a wireless USB dongle using customized acquisition software, OpenBCI GUI at a sampling frequency of 250 Hz.

### Experimental in vivo validation

Ethical approval was granted by the University of Auckland Animal Ethics Committee and the International Guiding Principles for Biomedical Research Involving Animals were followed. The protocols of animal preparations and recordings were similar to previously established experimental protocols [43]. A total of ten healthy cross-breed weaner pigs (weight 44.5 ± 7.6 kg) were housed individually and fasted overnight before the experiments to avoid compounding effects of ingestion. Each subject was induced with 6% Voluven saline (Fresenius Kabi Freeflex, USA) and general anesthesia with Zoletil (Tiletamine HCl 50 mg mL^−1^ and zolazepam HCl 50 mg mL^−1^). After induction, the subject was maintained with isoflurane (2.5–5%) with an oxygen flow of 400 mL within a closed-circuit anesthetic system. Vital signs (blood pressure, heart rate, and temperature) were monitored continuously throughout the experiments. The femoral artery was cannulated in the right limb for blood pressure. A rectal thermometer was pused to measure temperature. Endotracheal intubation was performed using Normocap 200 Oxy to monitor O_2_, CO_2_ and N_2_O levels. The subjects were maintained in a normal physiological range during the experiments by a respiration pump (Harvard Apparatus, co, Inc, USA) and a heating lamp.

The simultaneous recordings of cutaneous EGG and gastric slow waves were generally performed as the first study in a series of studies or during a period of stable signals to maximize the use of each subject and to avoid potential compounding effects of other experimental manipulations. Following light skin preparation of the abdominal region using Nuprep (Weaver and Company, Aurora, CO, USA), a mid-line laparotomy was performed to access the stomach of the subjects for serosal HR mapping. A commercial flexible printed circuit (FPC) electrode array (up to 256 channels at 4 mm inter-electrode spacing in a 16 × 16 configuration) (FlexiMap, New Zealand) was gently positioned onto the corpus of the stomach to record gastric slow waves with warm saline-soaked gauze packs overlaid on the electrodes to hold them in place. The DRL and CMS electrodes were placed on the right and left hind legs, respectively. The FPC array was validated for extracellular recordings with inhibition of contractions to demonstrate adequate signal with and without movement artifacts, which were suppressed using nifedipine [22, 23]. The incision was sutured closed for the duration of the experiment. The two versions of cutaneous EGG electrode arrays were then placed on the abdomen one at the time, positioned directly above the location of the stomach and oriented along the longitudinal axis of the stomach. Each recording was approximately 15–20 min to capture potential variations in gastric slow waves.

Each internal FPC array was connected to an ActiveTwo acquisition system (BioSemi, Amsterdam, Netherlands) via a 32-way ribbon cable, and the ActiveTwo was then connected to a recording laptop via a fiber-optic to USB connection. Data were acquired in Biosemi software (written in Labview v8.2, National Instruments, TX, USA) at a sampling frequency of 512 Hz. The Biosemi system was synched to the OpenBCI system to the nearest second aligning the start of recording between the two systems, using a trigger channel on the Biosemi system to indicate the start of the EGG recording.

### HR mapping and EGG signal processing

**Fig. 6:**
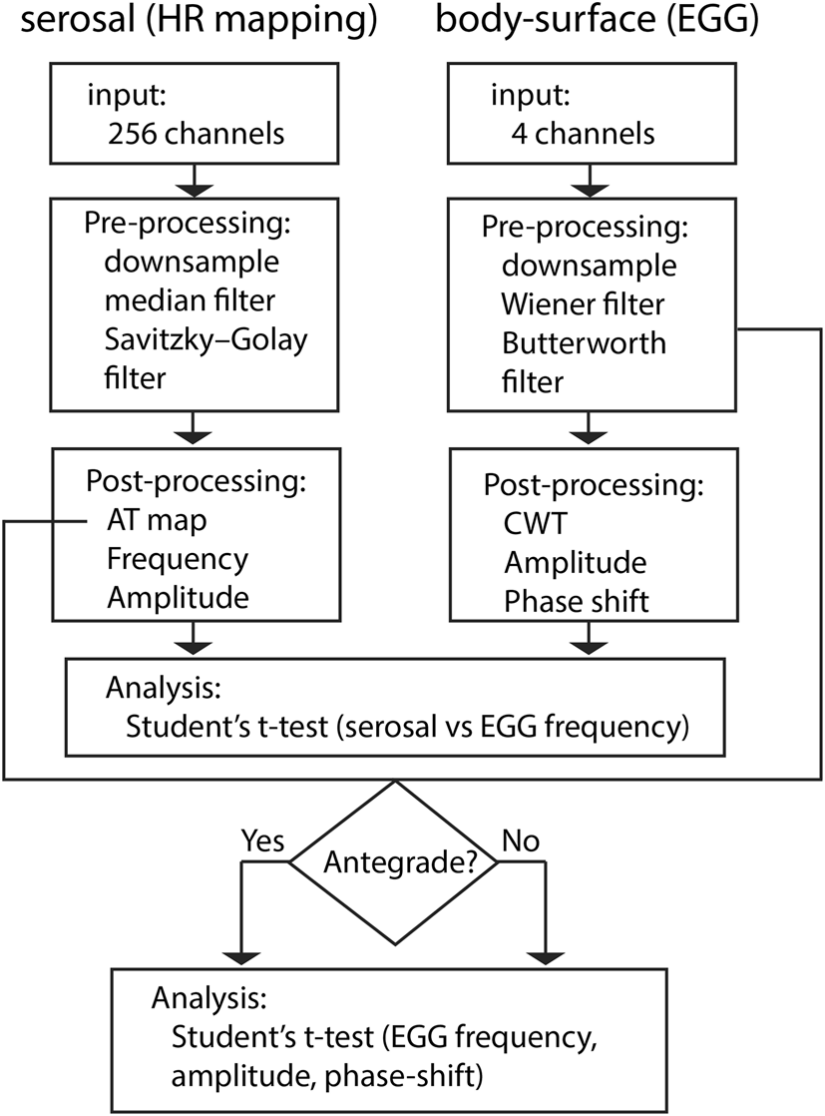
The flowchart of the experimental signal processing and analysis for the serosal (left) and cutaneous EGG recordings (right), starting from the inputs to the signal processing, analysis and outputs

Signal processing and analysis were performed in MATLAB (R2019a, MathWorks, Natick, MA, USA). The general workflow of signal processing and analysis is presented in Fig. 6.

The serosal recordings were processed in the Gastrointestinal Electrical Mapping Suite (GEMS v3.0) [46]. A median filter was used to remove the baseline drift and a Savitzky–Golay filter with an order of 9 and window width of 2 s was used to filter the signals. The activation time of each slow wave was automatically marked then grouped into discrete wavefronts (cycles), followed by manual review and correction. Regular antegrade propagation was classified as normal slow waves from the proximal corpus to the antrum, as defined in a previous baseline study [43]. Spatial dysrhythmias were defined as directional deviations from the typical antegrade propagation. The average amplitudes and frequency were quantified for every cycle of analyzed slow waves [46]. A separate one-way ANOVA was performed to compare amplitude and frequency measures of antegrade, retrograde and colliding wavefronts based on the classified serosal data. A follow-up Tukey interval analysis was performed if significance was found.

The cutaneous EGG signals from each channel were processed based on the PREP pipeline [47], which is a preprocessing standard designed for large-scale EEG analysis. Specifically, a moving median filter was applied, and then the signals were iteratively down sampled to 2 Hz with a low-pass filter up to 1/4 of the Nyquist frequency at each iteration. The final processing step involved applying a Weiner filter to remove baseline drift and a second order low-pass Butterworth filter with a cutoff frequency of 9 cpm. A continuous wavelet transform (CWT) using a Morse wavelet was applied to the filtered EGG signals to identify the change in frequency over time. The minimum and maximum scales are determined automatically based on the energy spread of the wavelet in frequency and time. Gastric slow waves in the filtered EGG signals were identified by the peak of each signal, followed by manual review and correction of the grouped slow wave events. Amplitude was calculated from each identified slow wave event in the filtered EGG and the average phase-shift was calculated between each neighboring EGG channel and classified based on the serosal activation profile.

Correlation between the serosal recordings and cutaneous EGG was analyzed by performing the Student’s *t*-test of the frequencies of slow waves from the serosal recordings and the dominant frequencies detected using CWT. In addition, amplitude, frequency, and phase-shift from EGG signals were also individually compared between periods of normal and abnormal slow waves using the Student’s *t*-test. A *p*-value of less than 0.05 was deemed to be statistically significant.

## Data Availability

The datasets used and/or analyzed during the current study are available from the corresponding author on reasonable request.

## Abbreviations

HR: High-resolution
EGG: Electrogastrography/electrogastrogram
CPM: Cycles per minute
opamps: Operational amplifiers
